# Vaping-Related Mobile Apps Available in the Google Play Store After the Apple Ban: Content Review

**DOI:** 10.2196/20009

**Published:** 2020-11-13

**Authors:** Meredith C Meacham, Erin A Vogel, Johannes Thrul

**Affiliations:** 1 Department of Psychiatry and Behavioral Sciences University of California San Francisco San Francisco, CA United States; 2 Stanford Prevention Research Center Department of Medicine Stanford University Stanford, CA United States; 3 Department of Mental Health Johns Hopkins School of Public Health Baltimore, MD United States

**Keywords:** vaping, mobile apps, nicotine, cannabis

## Abstract

**Background:**

In response to health concerns about vaping devices (eg, youth nicotine use, lung injury), Apple removed 181 previously approved vaping-related apps from the App Store in November 2019. This policy change may lessen youth exposure to content that glamorizes vaping; however, it may also block important sources of information and vaping device control for adults seeking to use vaping devices safely.

**Objective:**

Understanding the types of nicotine and cannabis vaping–related apps still available in the competing Google Play Store can shed light on how digital apps may reflect information available to consumers.

**Methods:**

In December 2019, we searched the Google Play Store for vaping-related apps using the keywords "vape" and "vaping" and reviewed the first 100 apps presented in the results. We reviewed app titles, descriptions, screenshots, and metadata to categorize the intended substance (nicotine or cannabis/tetrahydrocannabinol) and the app’s purpose. The most installed apps in each purpose category were downloaded and evaluated for quality and usability with the Mobile App Rating Scale.

**Results:**

Of the first 100 apps, 79 were related to vaping. Of these 79 apps, 43 (54%) were specific to nicotine, 3 (4%) were specific to cannabis, 1 (1%) was intended for either, and for the remaining 31 (39%), the intended substance was unclear. The most common purposes of the apps were making do-it-yourself e-liquids (28/79, 35%) or coils (25/79, 32%), games/entertainment (19/79, 24%), social networking (16/79, 20%), and shopping for vaping products (15/79, 19%). Of the 79 apps, at least 4 apps (5%) paired with vaping devices to control temperature or dose settings, 8 apps (10%) claimed to help people quit smoking using vaping, and 2 apps (3%) had the goal of helping people quit vaping.

**Conclusions:**

The majority of vaping-related apps in the Google Play Store had features either to help users continue vaping, such as information for modifying devices, or to maintain interest in vaping. Few apps were for controlling device settings or assisting with quitting smoking or vaping. Assuming that these Google Play Store apps were similar in content to the Apple App Store apps that were removed, it appears that Apple’s ban would have a minimal effect on people who vape with the intention of quitting smoking or who are seeking information about safer vaping via mobile apps.

## Introduction

### Background

The emergence of vaping, or e-cigarette technology, whereby a liquid solution is heated in a portable device until aerosolized and then inhaled, has been greeted with both promise and alarm [1-4]. In response to concerns about lung injury associated with vaping [5] and the increasing prevalence of youth vaping [6,7], Apple removed previously-approved vaping-related apps from its App Store (for iOS) in November 2019 [8], but the competing Google Play Store (for Android) did not remove vaping-related apps. Vaping-related apps may negatively influence youth by glamorizing vaping; however, some apps may benefit adults who use tobacco by providing cessation assistance or harm reduction information. The net balance of benefits and drawbacks from vaping-related apps likely depends on app content.

### Vaping Nicotine

Vaping nicotine may be less harmful than smoking combustible cigarettes [9] and may be a potential tool in helping people quit smoking [4,10,11]. Yet, alongside this potential is limited scientific evidence about the efficacy of nicotine vaping in promoting and maintaining tobacco-smoking cessation, as well as mounting concerns about a youth vaping epidemic [12,13]. Evidence to date appears to be mixed and context-dependent regarding whether e-cigarettes help adults quit [10] with concerns that the amount of nicotine in popular vaping products is too high and that people who quit smoking by vaping may return to smoking [1].

E-cigarettes are currently the most popular tobacco product among US youth, with an estimated 1 in 5 US high-school seniors reporting having vaped nicotine in the previous month, and 1 in 4 reporting having vaped nicotine- or flavor-containing e-liquids in 2018 [6]. A higher past-month prevalence of vaping was reported in the 2019 National Youth Tobacco Survey, with more than 1 in 4 US high-school students using e-cigarettes [7]. Although adolescents are smoking fewer cigarettes per day [14], and smoking has become less prevalent [15] in recent years, there are also reports of greater nicotine dependence among adolescents and young adults who vape prefilled pods (cartridges) [16], and a body of research indicating harm to the developing brain from nicotine [17]. In response, national and local governments have taken steps to propose or enact restrictions (or bans) on e-cigarette sales [18,19] and flavored vaping products [20].

### Vaping Cannabis

Similarly, vaping cannabis extracts or flower is believed to be less harmful than inhaling combusted cannabis [21-23]. Vaping is an increasingly popular method of cannabis consumption in the United States [24-26] although cannabis remains illegal in many US states and most countries. Both formal and informal cannabis product markets now offer a wide array of ways to consume tetrahydrocannabinol (THC), including vaping products [2,27]. The legalization of cannabidiol from hemp products in the United States in 2018 has led to an increase in interest and availability of cannabidiol-containing products [28] some of which can be vaped and do not need to be legally purchased from a licensed dispensary. Many vaping products with both THC and cannabidiol are sold online with limited regulation, and published product testing studies have found that tested cannabinoid content was often higher or lower than labeled content [29,30]. While Canada legalized cannabis nationally in October 2018, extracts and edibles were not legal until October 2019, due to additional difficulties in regulation.

Against this background, in the spring and summer of 2019, cases of acute lung injury related to vaping began to be reported in the United States. This new illness, named electronic or vaping product use associated lung injury (EVALI), ultimately caused at least 52 deaths and over 2600 hospitalizations by December 2019 [5]. Most of these patients were young adults who were previously healthy and reported a gradual onset of respiratory, constitutional, and gastrointestinal symptoms. Most—though not all—patients reported having used e-cigarette or vaping products containing THC. The most likely culprit was identified as vitamin E acetate, used as a bulking agent in primarily THC-containing vaping products, often procured in a state where cannabis was still illegal or from an unlicensed seller [31-34]. People who vape cannabis or nicotine may turn to mobile apps for guidance on safe use.

### Google Play Store Versus Apple iOS App Store

Mobile phones and mobile apps are increasingly used for accessing health and safety information about many topics [35,36], including substances. Apps are most used by younger populations with higher income and education [37]. The Google Play Store for Android and the Apple App Store for iOS are the 2 major mobile app platforms and marketplaces for digital apps, with 2.6 million available apps on Google Play and 1.8 million apps available on the Apple App Store in early 2020 [38]. Many popular apps have versions for both platforms. Both app stores have content, technical, and stylistic guidelines that developers must follow in order to have their app approved; violations can be grounds for removal of a previously approved app. It is generally believed that getting approval from the Apple App Store is more difficult than getting approval from the Google Play Store [39], with Apple App Store apps often viewed as higher quality and less likely to be free to use [40].

Both app stores specifically address tobacco and cannabis content in their developer guidelines (Table 1). Google Play Store’s substance-related content guidelines prohibit apps “facilitating the sale” of tobacco, marijuana, alcohol, or illegal drugs, or those “depicting or encouraging” use by minors [41]. The Apple App Store guidelines prohibit apps that “encourage consumption of tobacco and vape products, illegal drugs, or excessive amounts of alcohol,” particularly those encouraging minors [42]. Facilitating sale of these substances is also not allowed [42].

**Table 1:** App Store Review Guidelines related to tobacco, vaping, and marijuana.

App store and section		Text quotation
**Apple App Store for iOS**		
	Safety	*1.4.3 Apps that encourage consumption of tobacco and vape products, illegal drugs, or excessive amounts of alcohol are not permitted on the App Store. Apps that encourage minors to consume any of these substances will be rejected. Facilitating the sale of marijuana, tobacco, or controlled substances (except for licensed pharmacies) isn’t allowed.* [42]
**Google Play Store for Android**		
	Illegal Activities	*We don’t allow apps that facilitate or promote illegal activities.* *Examples of common violations* *Facilitating the sale or purchase of illegal drugs or prescription drugs without a prescription.* *Depicting or encouraging the use or sale of drugs, alcohol, or tobacco by minors.* *Instructions for growing or manufacturing illegal drugs.* [41]
	Inappropriate Content—Marijuana	*We don't allow apps that facilitate the sale of marijuana or marijuana products, regardless of legality.* *Allowing users to order marijuana through an in-app shopping cart feature.* *Assisting users in arranging delivery or pick up of marijuana.* *Facilitating the sale of products containing THC (Tetrahydrocannabinol), including products such as CBD oils containing THC.* [41]
	Inappropriate Content—Tobacco & Alcohol	*We don't allow apps that facilitate the sale of tobacco (including e-cigarettes and vape pens) or encourage the illegal or inappropriate use of alcohol or tobacco.* *Depicting or encouraging the use or sale of alcohol or tobacco to minors.* *Implying that consuming tobacco can improve social, sexual, professional, intellectual, or athletic standing.* *Portraying excessive drinking favorably, including the favorable portrayal of excessive, binge or competition drinking.* [41]

### Apple iOS App Store’s Vaping App Ban

In response to health concerns about vaping devices (eg, youth nicotine use, lung injury) and calls for bans on nicotine flavors and vaping devices, Apple prohibited new vaping-related mobile apps from its iOS App Store in June 2019 and removed 181 previously approved vaping-related apps on November 15, 2019 [8,43]. Articles published on technology, vaping, and cannabis industry–affiliated websites in the following weeks decried this move by Apple, stating that the ban impacted device users’ ability to safely use their products [44], removed a resource that assists people with quitting smoking [45], and would have detrimental effects on innovation [45], and that the app store approval process was inconsistent [46].

At a time of escalating concern about vaping, Apple’s policy change may benefit public health by lessening youth exposure to content that glamorizes vaping. Exposure to vaping-related content online has been associated with greater intentions to vape [47] and greater likelihood of vaping [48] among youth. However, Apple’s restrictions may also block important sources of information and vaping device control for adults seeking to use vaping devices safely.

The content and evidence base of vaping-related apps is currently unknown, though previous reviews and content analyses have examined apps related to smoking cessation, cannabis, alcohol [49], and other substance use [50]. Smoking cessation apps are abundant, though few have been demonstrated to be evidence-based [51-55], and many have limitations with usability [56]. A review [57] of cannabis apps conducted in 2014 found that the most common content areas largely consisted of cannabis strain classification guides, factoids about cannabis, and games, but few apps addressed negative health effects of cannabis use. To gain insight into the potential positive and negative effects of removing vaping-related apps from app stores, characterization of the remaining vaping-related apps in the Google Play Store, the main competing source for mobile apps, is an imperative first step.

### Study Objective

We analyzed the top vaping-related mobile apps on the Google Play Store with respect to app characteristics, intended purpose, and provision of features and information for limiting the potential harms of smoking and vaping. To our knowledge, the Google Play Store did not enact any restrictions on vaping-related apps in response to EVALI. Within the context of government and industry regulatory policy changes surrounding vaping devices, understanding the types of apps available for mobile phones and tablets can shed light on how this form of digital media may reflect and influence information about nicotine and cannabis vaping available to consumers. Depending on their purposes and features, vaping-related apps could encourage or discourage vaping. Some apps may provide resources for adults seeking to switch to vaping from smoking or to use vaping devices safely, while others may negatively impact youth by glamorizing vaping. As such, the removal of vaping-related apps may have mixed effects on population health, depending on which types of content predominate.

## Methods

### Selection of Apps

A search for apps using the keywords *vape* and *vaping* was performed on the Google Play Store on December 17, 2019 from an IP address in the United States. The names and order of the first 100 apps displayed in response to the search query were recorded. Similar to previous studies reviewing apps, only the first 100 apps of 250 search results were examined, because it is unlikely that individuals would browse further for a desired app without refining their search terms [56-58]. We chose not to include *e-cigarettes* or *e-cigs* as search terms because of their infrequent use outside of academic and regulatory discourses.

Resulting vaping apps available in the Google Play Store served as a proxy for apps that may have been available in the Apple App Store prior to November 2019, as a list of removed apps was not publicly available. Thus, coding vaping-related apps in the Google Play Store provided insight into the types of the apps both currently available to Google Play Store customers and previously available to Apple App Store customers. A search for *vaping* and *vape* in the Apple App Store in December 2019, performed on both a Mac and an iPhone, yielded zero results on the Mac Store and yielded only apps related to quitting vaping or to vapor-like image effects in the iPhone app store.

### Rating of Apps

Three investigators (MM, EV, JT) each extracted app characteristics directly from the Google Play Store for one-third of the apps (33-34 apps per investigator). Extracted app characteristics included the developer name, content and age ratings, cost, average star ratings, number of reviewers, number of installations, last date updated, and URL. An app purpose coding guide assessed whether the app was relevant to vaping (yes or no), the intended substance (nicotine/tobacco, cannabis/THC, or unclear), and the purpose of the app.

The app purpose coding guide was initiated by MM and developed iteratively among the investigators. Any information on the app store webpage, including description and screenshots, was used for coding the app purpose and content. Apps could have multiple purposes. After initial coding, the investigators discussed tabulated results and aspects of the coding guide that were unclear, then refined the coding guide based on common patterns and any confusing aspects of the coding guide.

The second coding guide was then applied to 20% of the apps, which were triple coded. Each investigator coded 21 apps (7 recoded from their first pass, with 7 each from the 2 other coders). Unanimous agreement across all 3 investigators was then assessed for the app’s relevance to vaping (yes or no), intended substance (nicotine/cannabis/unclear), and each of 10 potential purpose categories (coils, e-liquids, mods, shopping, games, social, device, quitting smoking, quitting vaping, other). If an app was determined to be about something other than vaping, its purpose was not coded. Agreement before consensus was 19/21 (90%) for relevance to vaping, 12/17 (71%) intended substance, and 14-17/17 (82%-100%) for the purpose categories. Individual apps for which at least one investigator had a discordant code were discussed until consensus was reached.

A third and final coding guide was applied to the full list of apps. Of note, the coding guide was clarified so that apps referencing *heat-not-burn* were coded as vaping-related. While tobacco heat-not-burn devices are considered distinct from vaping devices, heat-not-burn of cannabis flower is often considered vaping, and neither involves combustion. Additionally, the group could not determine the purpose of 6 apps from the Google Play Store descriptions. These apps were downloaded and evaluated using additional information from the downloaded app itself.

The final 8 purpose categories were do-it-yourself (DIY) coils, DIY e-liquids, shopping, entertainment, social, device, quitting smoking, and quitting vaping. Summary percentages and means for app metadata and purposes were calculated.

### Selection and Evaluation of Downloaded Apps

Next, apps within each of the 8 purpose categories were ranked by total number of installs, and the top 2 to 5 apps per category were downloaded for review. Instead of ranking the most popular apps, we ranked apps by popularity within categories, so that apps with less overall popularity but potentially important purposes would be included. The number of apps selected per category varied due to ties in the reported number of installs. Because apps could have multiple categories, this procedure resulted in a list of 18 apps with 10 to 1 million downloads each. Three of these apps disappeared from the store before they could be downloaded for review; one could be replaced with a premium version of the same app. A total of 16 apps were downloaded onto 2 Samsung Galaxy Tab A tablets and a Google Pixel 2 smartphone.

A random selection (6/16, 38%) was reviewed by all 3 investigators. Discrepancies were discussed, and the coding guide was updated. The final coding guide included evaluations of whether the content of the downloaded app matched the purpose category (yes or no) and whether it had the following types of information (yes or no): information about harms of vaping (eg, lung injury, nicotine dependence), information about safer vaping or DIY device use (eg, how to prevent explosions), and information about harms of smoking (eg, nicotine dependence, cardiovascular harms, cancer risk). For apps coded as being intended for quitting smoking or quitting vaping, the presence of a tracking feature in the downloaded app (eg, tracking days without smoking or vaping, tracking money saved) was noted (yes or no). For apps coded as pairing with devices, the presence of features for tracking temperature, dosage, or device locking was noted (yes or no). Differences between investigators were discussed until consensus was reached.

Finally, the Mobile App Rating Scale (MARS) was applied to all 16 downloaded apps. The MARS is 23-item multidimensional measure for rating the quality of mobile health apps, with 5 subscales in the areas of engagement (5 items), functionality (4 items), aesthetics (3 items), information (7 items, including affiliation of developer), and subjective rating (4 items) [59]. Subjective rating was not applied as these items involve hypothetical personal use. Each item was rated on a scale of 1 to 5, and ratings that differed by more than 2 points between investigators (eg, ratings of 1 vs 4 or 2 vs 5) were discussed. Investigators could adjust their ratings after discussion before averaging scores and did so 7 times across the 5 discussed apps. Some information items were rated as not applicable (N/A) and were excluded from average score calculations. For example, the item about meeting goals would be rated as N/A if the app purpose was not related to quitting smoking or vaping, and the visual information item would be rated as N/A if the app only contained text. Other smaller discrepancies were averaged without further discussion. Average ratings were calculated for each of the 4 subscales (engagement, functionality, aesthetics, information) and then averaged across the 3 investigator ratings. A final score was averaged for all downloaded apps and within each category.

## Results

### Overview of Vaping Apps

Of the top 100 apps captured by our search in December 2019, 79 (79%) were determined to be about vaping and were coded for purpose and other features. It was determined that 15 of the 21 apps not about vaping referred to cigarette smoking or only smoking cessation. An additional 6 apps could not be found again during content coding in January 2020 and February 2020 and were removed from the analysis. There were 4 sets of apps with both free and pro versions. The pro version typically cost money to download and did not have advertisements or had additional features. All app descriptions were in English, though some apps appeared to have content in other languages.

Of the 79 apps, most apps were free (60, 76%) or free with in-app purchases (13, 16%); 6 apps cost between US $0.99 and US $6.99 to download. Over half the apps had in-app advertisements (45, 57%). Only one-third of apps (36, 33%) were rated as Mature 17+, while the rest were rated as Teen (13, 16%) or Everyone (40, 51%) (Table 2).

There were 15 different Google Play Store–provided categories displayed with the app description, with the most common being tools and lifestyle, followed by health and fitness, simulation, and social. As one indicator of popularity, app downloads or installations ranged from at least 10 installs to over 1 million installs, with the first 10 apps presented in the search having at least 10,000 installs. As another set of popularity indicators, the average for the 68 apps with ratings was 4.0 stars, with a range of 2.5-5.0 stars by an average of 770 raters.

Investigator-coded app purposes are described in Table 2. The most common investigator-coded app purposes were creating DIY vaping e-liquids (28/79, 35%) and coils (25/79, 32%); 16 apps (16/79, 20%) were coded as for both creating coils and e-liquids. The next most common purposes were entertainment or games (19/79, 24%), social networking with other app users (16/79, 20%), and shopping (15/79, 19%). Social and shopping also tended to be co-occurring purposes (10/79, %). Finally, apps to help people quit smoking (8/79, 10%), directly control vaping devices (4/79, 5%), and quit vaping were less common (2/79, 3%). Out of 8 apps with quitting smoking features (typically a “cigarettes avoided” widget), 7 were also coded as DIY e-liquid or coil purposes. The majority referred to vaping with nicotine (43/79, 54%). Few apps were intended for cannabis (3/79, 4%), and 1 app referred to both nicotine and cannabidiol from cannabis (1/79, 1%). The intended substance was unclear in the remaining apps (31/79, 39%) (Table 3).

Because apps could fall into multiple categories, we did not statistically test differences in star ratings. However, we noted that the highest rated apps by users were in the DIY e-liquids and quitting smoking and vaping categories, while the lowest were in the device category. The most popular apps by number of installations were in the DIY e-liquids, DIY coils, and entertainment categories.

**Table 2:** Overview of vaping apps on Google Play Store in December 2019 (N=79).

Characteristic		Value, n (%)
**Cost**		
	Price $0.99-$6.99	6 (8)
	In-app purchases	13 (16)
	Free	60 (76)
**Advertisements**		
	Yes	45 (57)
	No	34 (43)
**Age rating**		
	Everyone	40 (51)
	Teen	13 (16)
	Mature 17+	26 (33)
**Categories**		
	Tools	25 (32)
	Lifestyle	22 (28)
	Health and fitness	5 (6)
	Simulation	4 (5)
	Social	4 (5)
	Art and design	4 (5)
	Other^a^	15 (19)
**Other content ratings**		
	Use of tobacco	7 (9)
	Use of drugs	3 (4)
	Drug reference	1 (1)
	Tobacco reference	1 (1)
	Language	1 (1)
	Violence, blood	1 (1)
**Total installs**		
	10 to 500	13 (16)
	1000 to 5000	36 (46)
	10,000 to 50,000	20 (25)
	100,000	8 (10)
	1,000,000	2 (3)
**Ratings**		
	Stars out of 5 (n=68), mean (SD)	4 (0.7)
	Raters (n=68), mean (SD)	770 (2607)
**Last updated**		
	2013-2016	13 (16)
	2017	11 (14)
	2018	24 (30)
	2019 (through November 14)	13 (16)
	November 15, 2019-February 29, 2020	18 (23)

^a^Other includes adventure, education, entertainment, libraries and demo, maps and navigation, medical, music and audio, personalization, productivity, and travel and local categories.

**Table 3:** Purpose and intended substance ratings for vaping apps on Google Play Store (N=79).

Category		Description	Apps, n (%)	With star ratings, n	Star rating, mean (SD)	Installations, n
**Purpose^a^**						
	DIY^b^ e-liquids	Has features for creating e-liquids (eg, calculators with nicotine and propylene glycol or vegetable glycerin inputs, e-liquid recipes)	28 (35)	26	4.18 (0.60)	500+ to 1,000,000+
	DIY coils	Has features for designing coils for DIY mods (eg, Ohm’s law calculators)	25 (32)	25	4.01 (0.68)	500+ to 1,000,000+
	Entertainment	Simulations, wallpapers, games	19 (24)	14	3.74 (0.65)	100+ to 1,000,000+
	Social	Has features for connecting with other app users or people who vape, including the ability to review products	16 (20)	15	4.03 (0.79)	50+ to 100,000+
	Shopping	For finding products or stores that sell vaping devices or products or e-liquids	15 (19)	13	3.92 (0.93)	50+ to 100,000+
	Quitting smoking	For helping people quit smoking (using vaping or not); may include a tracking feature oriented around not using cigarettes	8 (10)	8	4.06 (0.52)	500+ to 10,000+
	Device	Pairs with and has features for modifying a device	4 (5)	2	2.65 (0.07)	100+ to 10,000+
	Quitting vaping	For helping people quit vaping	2 (3)	1	4.10 (N/A^c^)	10+ to 1000+
**Substance**						
	Cannabis	N/A	3 (4)	2	3.55 (1.34)	100+ to 10,000+
	Nicotine	N/A	43 (54)	38	3.97 (0.62)	10+ to 1,000,000+
	Unclear	N/A	31 (39)	26	4.07 (0.78)	50+ to 100,000+
	Both	N/A	1 (1)	1	4.70 (N/A)	10,000+

^a^Apps could have multiple purposes.

^b^DIY: do-it-yourself.

^c^N/A: not applicable.

### Downloaded Apps

The majority of downloaded apps (14/16, 88%) matched the descriptions in the app store (Table 4). The exceptions were that 1 app coded as having quitting smoking features did not have any such features, and 1 app that was coded as not having shopping features did, in fact, have links to shopping through the app. Few apps had information about harms of vaping (4/16, 25%), safer vaping (3/16, 19%), or harms of smoking (2/16, 13%). When they did have such information, it was often difficult to find. Of note, 2 of the entertainment apps had a feature where an avatar would cough when vaping too much, which may normalize moderation in vaping; 5 apps had tracking features, which mainly recorded days passed since a user-provided quit-smoking date; and 2 of the apps with tracking features also displayed money saved and health benefits. Both device apps appeared to have temperature controls, but did not have dosage or locking settings visible, though these may have become apparent once paired with a device.

Overall, the 16 downloaded apps had a mean MARS score of 3.63, with a highest mean subscore for functionality (MARS score: mean 4.13) and lowest mean subscore for engagement (MARS score: mean 3.36). Within the subtypes of purposes, the highest mean MARS scores were for social, shopping, and device apps, and the lowest mean scores were for the quitting smoking and DIY coils apps.

**Table 4:** Ratings of downloaded vaping apps (N=16).

Characteristic and subcharacteristic		Value
**Purpose, n (%)**		
	Matches coded purpose	14 (88)
**Information, n (%)**		
	Harms of vaping	4 (25)
	Safer vaping	3 (19)
	Harms of smoking	2 (13)
**Behavior change, n (%)**		
	Tracking feature	5 (31)
**Device (n=2), n (%)**		
	Temperature	2 (100)
	Dosage	0 (0)
	Locking	0 (0)
**MARS^a^ (out of 5, all downloaded apps), mean (range)**		
	Engagement	3.36 (2.80-4.20)
	Functionality	4.13 (3.08-4.58)
	Aesthetics	3.40 (2.22-4.50)
	Information	3.61 (2.50-4.83)
	Summary	3.63 (2.77-4.47)
**MARS (out of 5, by purpose category)^b^, mean (range)**		
	DIY^c^ e-liquids (n=5)	3.54 (3.16-4.04)
	DIY coils (n=3)	3.44 (3.16-3.89)
	Entertainment (n=3)	3.66 (3.00-4.47)
	Social (n=3)	4.22 (4.04-4.47)
	Shopping (n=2)	4.09 (4.04-4.15)
	Quitting smoking (n=2)	3.08 (2.89-3.49)
	Device (n=2)	4.04 (3.93-4.15)
	Quitting vaping (n=2)	3.93 (3.80-4.06)

^a^MARS: Mobile App Rating Scale.

^b^Top 2 to 5 apps per category by number of installations; categories with 3 to 5 apps had ties in the number of installations.

^c^DIY: do-it-yourself.

## Discussion

This study examined the content of the first 100 mobile apps on the Google Play Store using *vaping* and *vape* as search terms 1 month after Apple’s ban on vaping-related apps, which was enacted in response to concerns about youth nicotine vaping and EVALI (a lung injury syndrome linked to an additive to cannabis vaping products). Of 79 apps determined to be related to vaping, over half were related to nicotine, while only a few were for cannabis, and the rest were unclear in intended substance. The most popular app content, with respect to both number of installations (several with over 1 million) and percentage of these 79 vaping apps, was creating DIY liquids (28/79, 35%) and DIY coils (25/79, 32%), with 20% (16/79) in both categories (37 total). This may reflect a strong interest in DIY hobbies in vaping culture. DIY may allow users to control the customization process, play with novelty, save money, and achieve higher nicotine concentrations [60,61]. Overall, the main purposes of the majority of these vaping-related apps on the Google Play Store were to help people continue to vape nicotine.

Apps that had features to support quitting smoking or vaping were relatively rare (8/79,10%; 2/79, 3%; respectively). Most apps that supported smoking cessation encouraged users to quit smoking by switching to vaping. These apps also contained other features to promote or facilitate vaping, such as e-liquid recipes. The 2 apps for quitting vaping that were downloaded had above average MARS scores but relatively few installations, while the 3 apps for quitting smoking that were downloaded had below average MARS scores and low subscores on aesthetics and information. This points to the need for apps to promote vaping cessation using evidence-based behavior-change information and strategies, along with engaging usable interfaces.

There were also few apps that paired with devices (4/79, 5%), and these device apps were mainly for cannabis. Devices that pair with apps using Bluetooth technology are more expensive, which may explain their lower popularity in terms of installs. Features available in the downloaded device apps indicated that the user could control temperature [62], which may limit throat and lung damage, but not dosage, and there were no locking controls for users with children or those wishing to moderate their use.

Given current concerns about youth vaping, the findings that over half the apps had no age controls and that a large proportion of apps without age controls was in the DIY categories (24/37, 65%) are especially concerning. A smaller proportion of entertainment apps had no age controls (5/19, 26%), though many of the apps with age controls were set at Teen. Age controls may allow parents who utilize family controls to restrict their children’s access to these apps.

Of the popular apps that were downloaded and reviewed in-depth, few apps presented information about the harms of vaping or smoking or included information about safer vaping. Information about harms of smoking consisted of articles comparing the harms of smoking (eg, combustion and cancer-causing ingredients) to vaping. One app had a widget for tracking “days without smoking” that included “gained days of life” and “avoided radiographs” calculations.

Information presented about harms of vaping included acknowledgments of the importance of moderating nicotine intake, the addictiveness of nicotine, or harms of vaping in front of children. The 2 downloaded apps that were intended to help people quit vaping both included links to news stories about young adults with EVALI and articles about concerns with youth vaping and the intentions of vaping companies. Safer vaping information included recommendations about coil and battery materials, causes of e-cigarette explosions, and cautions against mixing e-liquids incorrectly. It should be noted that in 2 entertainment apps with simulated vaping games, the vaping avatar would cough audibly when they “inhaled” a large amount of vapor, which could be seen as encouraging moderation in use. Several of these apps included a tracking feature that displayed the number of days since quitting smoking and the number of cigarettes avoided. Self-monitoring is an important component of a smoking cessation plan but is likely insufficient by itself [63]. In addition to a need for apps on the Google Play store that assist people with quitting vaping, study results indicate a need for informational apps to better describe the pros and cons of vaping.

Assuming that these Google Play Store apps were similar in content to the Apple App Store apps that were removed, it appears that Apple’s ban would have had a minimal effect on people who vape with the intention of quitting smoking or who are seeking information about safer vaping. Nevertheless, the decision to remove the vaping-related apps appears to have been taken by Apple in response to rising EVALI cases, which were primarily attributed to cannabis oil additives, rather than nicotine liquids [33,34]. There appeared to be little publicly available information detailing how apps were determined to be removed, echoing other calls for increased transparency and additional research regarding allowed app platform content and other issues like privacy [39,64]. Future research should explore other cases touching on the who and how of regulation of apps related to controversial health-behavior for which there is not yet a consensus among health experts. Future research should also examine more explicitly the relationship between vaping app use and vaping behaviors.

There were several limitations to this study. First, not all apps that came up in the initial search were reviewed, though most app users would likely not browse more than 100 apps without refining their search. Additionally, the app store gets updated continuously, and a search on a different date may present different results. Indeed, several apps were no longer available a few weeks after the initial search. While the number of installations was recorded for each as a signal of popularity, people may download an app and not use it at all or only use it a limited number of times. Only 1 of the reviewed apps was also available on the iPhone App Store, but it is unclear which of the apps we reviewed were removed from or denied approval in the Apple App Store. Although we only coded Google Play Store apps, a search for *vaping* in the mobile Apple App Store in December 2019 confirmed that the remaining apps were related to quitting smoking or quitting vaping or were unrelated to vaping behavior.

Based on this review of vaping-related apps in the Google Play Store, it appears that the Apple vaping app ban would have had a minimal effect on adults seeking to switch away from smoking or seeking to vape more safely. Most vaping-related apps in the Google Play Store were for purposes related to continuing vaping and had limited age-based access restrictions. Few apps were for controlling device settings, assisting with quitting smoking or vaping, or disseminating information about safer vaping.

## Abbreviations

DIY: do-it-yourself
EVALI: electronic or vaping product use associated lung injury
IP: internet protocol
MARS: Mobile App Rating Scale
THC: tetrahydrocannabinol
URL: uniform resource locator
